# Clinical Efficacy Study of Escitalopram Combined With Low-Frequency Repetitive Transcranial Magnetic Stimulation in Patients With Post-Ischemic Stroke Depression and Anxiety

**DOI:** 10.62641/aep.v54i4.2250

**Published:** 2026-08-15

**Authors:** Yan Long, Qingxian Wei, Xiujin Deng, Jiezhi Wei, Ailing Jiang, Qing Guo, Yuanqiu Zhong

**Affiliations:** ^1^Department of Ultrasound Medicine, Jiangbin Hospital of Guangxi Zhuang Autonomous Region, 530021 Nanning, Guangxi, China; ^2^Department of Gastroenterology, Jiangbin Hospital of Guangxi Zhuang Autonomous Region, 530021 Nanning, Guangxi, China; ^3^Department of Neurology, Jiangbin Hospital of Guangxi Zhuang Autonomous Region, 530021 Nanning, Guangxi, China

**Keywords:** escitalopram, transcranial magnetic stimulation, ischemic stroke, depression, anxiety

## Abstract

**Background:**

To retrospectively analyze the clinical efficacy of escitalopram combined with low-frequency repetitive transcranial magnetic stimulation (LF-rTMS) in treating anxiety and depression in ischemic stroke patients.

**Methods:**

Eighty ischemic stroke patients with concurrent depression and anxiety who received treatment at Jiangbin Hospital of Guangxi Zhuang Autonomous Region from January 2025–December 2025 were retrospectively collected. Patients were divided into a conventional treatment group and a combined treatment group, with 40 patients in each group. The conventional treatment group received routine treatment combined with oral escitalopram, while the combined treatment group received the same treatment as the conventional treatment group, combined with LF-rTMS. To evaluate clinical efficacy, Hamilton Depression Rating Scale (HAMD) and Hamilton Anxiety Rating Scale (HAMA) scores were collected before and at the end of treatment in both groups. Transcranial ultrasound imaging of the brainstem raphe (BR) region was also performed, and adverse reactions during treatment were recorded.

**Results:**

The total effective rate in the combined treatment group was significantly higher than that in the conventional treatment group. After adjusting for baseline scores, post-treatment HAMD and HAMA scores in the combined treatment group were significantly lower than those in the conventional treatment group. The proportion of patients with abnormal echogenicity decreased in both groups, and the number of patients with abnormal echogenicity in the combined treatment group was less than that in the conventional treatment group. There was no statistically significant difference in the incidence of adverse reactions between the two groups.

**Conclusions:**

Escitalopram combined with LF-rTMS can effectively alleviate anxiety and depression in ischemic stroke patients and show beneficial changes in BR echogenicity as an exploratory imaging finding. It may serve as a potential and promising treatment option for post-ischemic stroke depression and anxiety, although further validation is needed.

## Introduction

Ischemic stroke is a disease caused by sudden cerebrovascular lesions that result in focal or diffuse brain damage. It is characterized by high incidence, high recurrence rate, and high disability and mortality rates, and is one of the leading causes of death among Chinese residents [1, 2]. At the same time, the prevalence of psychological disorders including anxiety and depression among stroke patients can reach 30%–50%. Specifically, the prevalence of post-stroke anxiety is approximately 19.3%–30.8%, and that of post-stroke depression is about 31%–49.1%, and the incidence is significantly higher than in the general population. These psychological disorders have become a common issue affecting the overall rehabilitation of patients [3]. It often manifests as post-ischemic stroke depression and anxiety, with low mood, fatigue, loss of interest, and lack of energy as the main symptoms, which can lead to a significant decrease in the patient’s enthusiasm for actively cooperating with disease treatment and nursing care [4]. Therefore, exploring effective psychological management strategies is of great significance for improving the psychological state of post-ischemic stroke depression and anxiety patients and reducing the burden on patients’ families [5]. Effective psychological support and management can improve the emotional well-being of patients with depression and anxiety following ischemic stroke, enhance treatment adherence and participation in rehabilitation, thereby improving functional outcomes and quality of life, while also alleviating psychological stress in caregivers and reducing the need for long-term care [6]. Escitalopram is a selective serotonin reuptake inhibitor (SSRI) antidepressant, mainly used to treat depression and related mood disorders. It helps improve mood and relieve anxiety by regulating the level of the neurotransmitter serotonin in the brain, and has a high safety profile [7]. Transcranial magnetic stimulation (TMS) is a non-invasive technique based on Faraday’s law of electromagnetic induction. It uses an alternating magnetic field to stimulate brain nerves and generates a small current through electromagnetic induction to regulate neuronal excitability in order to restore the balance between excitation and inhibition functions in the brain [8, 9]. Its technical modes include single-pulse, double-pulse, and repetitive stimulation. Among them, the high-frequency mode of repetitive stimulation can enhance neural activity, while the low-frequency mode has an inhibitory effect [10]. Clinically, it is mainly used for depression, anxiety, schizophrenia, Parkinson’s disease, post-traumatic stress disorder, vegetative state, and post-ischemic stroke rehabilitation. It has unique value in the non-drug management framework system for neuropsychiatric diseases, and is especially suitable for patients who do not respond well to drug treatment, have contraindications, or seek synergistic effects [11]. Currently, there are clinical studies using escitalopram combined with repetitive transcranial magnetic stimulation (rTMS) to treat anxiety and depression, providing an effective way to treat [12]. However, existing studies mainly focus on the improvement of clinical symptoms, and there is still insufficient objective imaging evidence regarding the potential neurobiological mechanisms behind the combined treatment, especially the impact on the structure or function of key brain regions. In addition, the relationship between the clinical response to low-frequency repetitive transcranial magnetic stimulation (LF-rTMS) combined with escitalopram in post‑ischemic stroke depression and anxiety and brainstem raphe (BR) echo characteristics has not been fully elucidated, hindering the identification of objective imaging markers to guide the optimization and clinical application of this regimen. Therefore, this study aims to evaluate the therapeutic effects of escitalopram combined with LF-rTMS in patients with post-ischemic stroke depression and anxiety, and to explore changes in BR echogenicity as an exploratory neuroimaging observation. We used transcranial ultrasound to dynamically observe the changes in BR echogenicity characteristics, which may provide supportive clues for understanding the potential synergistic effect of the combined regimen, rather than verifying its mechanism of action. This study may provide a more objective clinical and imaging reference for optimizing the treatment of post-ischemic stroke depression and anxiety.

## Materials and Methods

### General Information

We retrospectively collected data from 80 ischemic stroke patients with depression and anxiety who received treatment at Jiangbin Hospital of Guangxi Zhuang Autonomous Region between January 2025–December 2025. They were divided into a conventional treatment group and a combined treatment group, with 40 patients in each group, according to the actual treatment plan. Inclusion criteria: (1) Meets the diagnostic criteria for ischemic stroke in the Chinese Guidelines for the Diagnosis and Treatment of Acute Ischemic Stroke 2018 [13], namely, based on the symptoms and signs of focal neurological deficits in acute onset (such as hemiplegia, aphasia, sensory disturbances, etc.), and after excluding cerebral hemorrhage and other non-vascular causes by head computed tomography or magnetic resonance imaging, the final clinical diagnosis is made by a neurologist in combination with the imaging results; (2) Age 18 to 75 years, gender not limited; (3) Post-ischemic stroke depression and anxiety were diagnosed by two independent senior psychiatrists according to the International Classification of Diseases, 11th Revision (ICD-11) [14], namely: significant depressive mood or loss of interest lasting for at least 2 weeks, accompanied by other symptoms such as changes in energy, sleep, or appetite. For anxiety, the core criteria included persistent, uncontrollable excessive anxiety lasting for several months, accompanied by obvious muscle tension or autonomic symptoms. The diagnosis was based on structured psychiatric interviews, clinical manifestations, and medical records, combined with Hamilton Depression Rating Scale (HAMD) and Hamilton Anxiety Rating Scale (HAMA) scores; (4) Clinically stable condition: at least 2 weeks after ischemic stroke onset, stable vital signs, no obvious progression of neurological deficits, and no acute cardiovascular or cerebrovascular events or severe complications within the past 2 weeks; (5) complete medical records, including treatment records, scale assessments and imaging data. Exclusion criteria: (1) severe heart, liver, kidney failure or other serious systemic diseases; (2) history of mental illness or long-term use of antipsychotic drugs; (3) contraindications to TMS, such as intracranial metal implants, history of epilepsy, pacemaker, etc.; (4) allergy to escitalopram or contraindications; (5) Patients with severe cognitive impairment (Montreal Cognitive Assessment score < 10) [15] or inability to understand and cooperate with psychological scale assessments, which may compromise the accuracy of HAMD and HAMA results.

### Treatment Methods

Both groups of patients received routine in-hospital treatment, including antiplatelet or anticoagulant therapy as indicated, statin therapy to stabilize plaques, and strict control of blood pressure and blood sugar as secondary prevention measures. They underwent daily systematic neurorehabilitation guided by rehabilitation therapists, including physical therapy and occupational therapy. Targeted assessments and management were provided for patients with speech or swallowing difficulties. Medical staff routinely conducted health education related to ischemic stroke and mood disorders, and provided unstructured emotional reassurance and psychological support during daily ward rounds. The conventional treatment group received only routine treatment plus escitalopram; the specific content of routine treatment was clearly defined as follows: basic ischemic stroke medical management (standardized control of blood pressure, blood glucose, and blood lipids to prevent recurrence), standardized rehabilitation programs including physical therapy (limb function training, gait training, and muscle strength training) and occupational therapy (daily living ability training, such as dressing, eating, and bathing training), and symptomatic support treatment (nutritional support, prevention of pulmonary infection and other complications).

The combined treatment group received LF-rTMS treatment in addition to the treatment regimen of the conventional treatment group [16]. During treatment, patients sat comfortably with their head stabilized. First, the resting motor threshold (RMT) was determined as the minimum intensity that elicited a visible motor twitch in the contralateral distal wrist muscles, using single pulses over the left motor cortex. LF-rTMS was then delivered using a rTMS device with a figure-of-eight coil. The stimulation target was the right dorsolateral prefrontal cortex (rDLPFC), which was localized to the F4 scalp position defined by the international 10–20 Electroencephalogram (EEG) system via a structured, validated measurement with a measuring tape [17]. The coil was placed tangentially to the scalp at a 45° angle toward the midline to ensure optimal stimulation of the underlying rDLPFC. Stimulation parameters were set as follows: frequency 1 Hz, intensity 120% of the RMT. The protocol consisted of 6 trains of 60-second duration (60 pulses per train), separated by 30-second inter-train intervals, consistent with the validated protocol described in the literature [16]. Each treatment session lasted a total of 8 minutes and 30 seconds, administered once daily, 5 days per week for 4 consecutive weeks, with a total of 20 sessions completed per patient. Patient tolerance and adverse reactions were closely monitored throughout the treatment period.

### Observation Indicators

Baseline assessments included gender, age, education level, history of hypertension, history of diabetes, previous stroke history, lesion location, time from ischemic stroke onset to enrollment, National Institutes of Health Stroke Scale (NIHSS) score for neurological deficit severity, as well as baseline severity categories of depression (HAMD) and anxiety (HAMA).

HAMD and HAMA scores of the two groups of patients before and after treatment were extracted from the medical record system [18]. At the same time, the detection results of the BR region of ischemic stroke patients by transcranial ultrasound imaging were extracted. The types, frequencies and severity of adverse reactions during treatment were extracted from nursing records and adverse event reports.

#### HAMD

The 17-item HAMD was used in this study. Items were scored on a standard 0–4 or 0–2 scale according to the original scale criteria. The total score was calculated as the sum of all items, with a range of 0–52 points. Higher scores indicate more severe depressive symptoms [18]. According to commonly used and validated clinical criteria, 0–7: no depression; 8–16: mild depression; 17–23: moderate depression; ≥24: severe depression [19]. The scale has good inter‑rater reliability, internal consistency, and structural validity, and is recognized as a reliable and widely used instrument for the assessment of depression.

#### HAMA

The 14-item HAMA was used. Each item was scored on a 0–4 scale, with a total score range of 0–56 points. Higher scores indicate more severe anxiety symptoms [18]. Severity classification: 0–6: no anxiety; 7–13: mild anxiety; 14–20: moderate anxiety; 21–28: severe anxiety; ≥29: extremely severe anxiety [20]. The scale has good reliability and validity and is widely used internationally for the evaluation of anxiety.

#### BR Detection

The BR echo was divided into four levels: Level 1, where the BR echo was not visible; Level 2, where the BR echo was interrupted or discontinuous; Level 3, where the BR echo was reduced; and Level 4, where the BR echo was continuous and normal [21]. (Echo abnormalities include disappearance, interruption, and weakening). The occurrence of adverse reactions was also recorded.

#### Adverse Reaction Classification

Based on the Good Clinical Practice for Drug Clinical Trials [22] and the Regulations on the Reporting and Monitoring of Adverse Drug Reactions [23], this study categorizes adverse reactions into the following two types according to their severity:

Serious adverse reactions refer to events that require emergency medical care and are clinically harmful at any dose, including: (1) death or life-threatening events; (2) hospitalization or prolonged hospitalization; (3) persistent or significant loss of function or disability; (4) congenital abnormalities or birth defects; (5) other important medical events [24].

Mild adverse reactions refer to reactions with mild symptoms that do not affect daily activities and usually do not require special treatment or only symptomatic treatment [25]. The main adverse reactions observed in this study include: (1) gastrointestinal symptoms (such as nausea, vomiting, dry mouth); (2) neurological symptoms (such as headache, dizziness, drowsiness, insomnia); (3) other common reactions (such as fatigue, excessive sweating, local scalp discomfort). All adverse reactions were recorded in detail and their relevance to treatment was analyzed to comprehensively assess treatment safety.

### Statistical Methods

Data were analyzed using SPSS 27.0 (IBM, Armonk, NY, USA). Normality was assessed using the Kolmogorov-Smirnov test, and homogeneity of variance was assessed using Levene’s test. Continuous variables with normal distribution were expressed as mean ± standard deviation (SD).

Baseline comparisons: Independent samples *t*-test was used for continuous variables; Pearson’s chi-square test (or Fisher’s exact test when expected cell frequency < 5) was used for categorical variables.

Within-group comparisons (pre- vs post-treatment): Paired *t*-test was applied for HAMD and HAMA scores; the Wilcoxon signed-rank test was applied for BR echogenicity grade.

Between-group comparisons (post-treatment): Analysis of covariance (ANCOVA) was used to compare post-treatment HAMD and HAMA scores, with baseline scores as the covariate, Partial eta-squared was reported as the effect size; the Mann–Whitney U test was applied for BR echogenicity grade.

For the comparison of adverse reaction incidence, Fisher’s exact test was used due to small expected frequencies.

A two-tailed *p* < 0.05 was considered statistically significant.

## Results

### Comparison of Baseline Characteristics Between the Two Groups

Baseline demographic, vascular risk factors, stroke-related and psychiatric characteristics were compared between the combined treatment group and the conventional treatment group. There were no statistically significant differences in gender, age, education level, hypertension, diabetes, previous stroke history, lesion location, time from stroke onset to enrollment, baseline NIHSS score, baseline HAMD severity, or baseline HAMA severity between the two groups (all *p* > 0.05).The baseline data were well balanced and comparable between groups, as shown in Table 1.

**Table 1.** **Comparison of general and baseline clinical characteristics between the two groups**.

Index		Combined treatment group ( n = 40 )	Conventional treatment group ( n = 40 )	χ^2^ / *t*	*p*
Gender, n (%)				χ^2^=0.833	0.361
	Male	14 (35.00)	18 (45.00)		
	Female	26 (65.00)	22 (55.00)		
Age ($\bar{x}$ ± s in years)		66.60 ± 9.45	67.50 ± 7.95	*t* = –0.516	0.608
Education level, n (%)				χ^2^ = 0.065	0.799
	Junior high school and below	29 (72.50)	30 (75.00)		
	Junior high school and above	11 (27.50)	10 (25.00)		
History of hypertension, n (%)		23 (57.50)	25 (62.50)	χ^2^ = 0.208	0.648
History of diabetes, n (%)		10 (25.00)	15 (37.50)	χ^2^ = 1.455	0.228
Previous stroke, n (%)		6 (15.00)	6 (15.00)	χ^2^ = 0.000	1.000
Lesion location, n (%)				χ^2^ = 0.059	0.971
	Left hemisphere	18 (45.00)	17 (42.50)		
	Right hemisphere	16 (40.00)	17 (42.50)		
	Bilateral / brainstem	6 (15.00)	6 (15.00)		
Time from stroke onset to enrollment, days		14.85 ± 4.62	15.30 ± 5.11	*t* = –0.412	0.681
NIHSS score at baseline		6.85 ± 2.40	7.10 ± 2.65	*t* = –0.441	0.660
Baseline HAMD severity, n (%)				χ^2^ = 0.051	0.821
	Moderate (17–23)	22 (55.00)	23 (57.50)		
	Severe (≥24)	18 (45.00)	17 (42.50)		
Baseline HAMA severity, n (%)				χ^2^ = 0.054	0.816
	Moderate (14–20)	26 (65.00)	25 (62.50)		
	Severe (21–28)	14 (35.00)	15 (37.50)		

HAMD, Hamilton Depression Rating Scale; HAMA, Hamilton Anxiety Rating Scale; NIHSS, National Institutes of Health Stroke Scale.

### Comparison of Treatment Efficacy Between the Two Groups of Patients

Before treatment, there were no significant differences in HAMD and HAMA scores between the two groups (*p* > 0.05). After adjusting for baseline values using ANCOVA, post-treatment HAMD and HAMA scores in the combined treatment group were significantly lower than those in the conventional treatment group (*p* < 0.001), as shown in Table 2.

**Table 2.** **Comparison of post-treatment HAMD and HAMA scores between the two groups (ANCOVA analysis)**.

Index	Group	n	Baseline (mean ± SD)	Post-treatment (Adjusted mean ± SE)	Between-group comparison (ANCOVA)
HAMD	Combined treatment	40	25.20 ± 3.05	10.32 ± 0.47***^#⁢#⁢#^	F(1, 77) = 126.58 *p* < 0.001 partial $\eta^{2}$ = 0.622
	Conventional treatment	40	24.85 ± 3.21	15.40 ± 0.50***	
HAMA	Combined treatment	40	21.95±3.12	9.87 ± 0.38***^#⁢#⁢#^	F(1, 77) = 118.72 *p* < 0.001 partial $\eta^{2}$ = 0.606
	Conventional treatment	40	22.10 ± 2.98	13.25 ± 0.41***	

Note: ***p < 0.001 compared with pre-treatment values within the same group (paired samples *t*-test). ^#⁢#⁢#^*p* < 0.001 compared with the conventional treatment group at the same time point (ANCOVA, adjusted for baseline scores). HAMD, Hamilton Depression Rating Scale; HAMA, Hamilton Anxiety Rating Scale; SD, standard deviation; SE, standard error; ANCOVA, analysis of covariance; partial $\eta^{2}$, partial eta-squared (effect size of ANCOVA model).

### Comparison of BR Echo Grade Distribution Before and After Treatment in the Two Groups of Patients

Before treatment, there was no significant difference in the distribution of BR echogenicity grades between the two groups (*p* > 0.05). After treatment, the BR grade distribution improved significantly in both the combined treatment group (*p* < 0.001) and the conventional treatment group (*p* = 0.004). Moreover, the combined treatment group exhibited a significantly better BR echogenicity grade distribution than the conventional treatment group at post-treatment (*p* = 0.028), as shown in Table 3.

**Table 3.** **Comparison of BR echo grade distribution before and after treatment in the two groups of patients [n (%)]**.

Group	Number of examples	Time point	Anomaly in echo (level 1–3)			Normal echo (Level 4)	*p* within-group comparison (Wilcoxon signed-rank test)	*p* between-group comparison (Mann–Whitney U test)
			Level 1 (Invisible)	Level 2 (Interruption)	Level 3 (Reduced)			
Combined treatment group	40	Before treatment	15 (37.50)	12 (30.00)	8 (20.00)	5 (12.50)	<0.001	0.028
		After treatment	10 (25.00)	6 (15.00)	2 (5.00)	22 (55.00)		
Conventional treatment group	40	Before treatment	14 (35.00)	13 (32.50)	9 (22.50)	4 (10.00)	0.004	0.877
		After treatment	12 (30.00)	9 (22.50)	6 (15.00)	13 (32.50)		

BR, brainstem raphe.

### Occurrence of Adverse Reactions in the Two Groups of Patients After Treatment

No serious adverse reactions occurred in either group during treatment. According to the classification in the Adverse Reaction Classification section, mild adverse reactions were categorized into gastrointestinal symptoms (nausea, dry mouth) and neurological symptoms (drowsiness, dizziness). In the combined treatment group, 5 patients (12.50%) developed mild adverse reactions, including gastrointestinal symptoms in 2 patients (5.00%) and neurological symptoms in 3 patients (7.50%). In the conventional treatment group, 4 patients (10.00%) had mild adverse reactions, including gastrointestinal symptoms in 2 patients (5.00%) and neurological symptoms in 2 patients (5.00%). All adverse events were mild and resolved spontaneously after symptomatic management. There was no statistically significant difference in the overall incidence of mild adverse reactions between the two groups (Fisher’s exact test, *p* = 1.000), as shown in Table 4.

**Table 4.** **Occurrence of adverse reactions [n (%)]**.

Group	Total occurrence	Gastrointestinal symptoms	Neurological symptoms
Combined treatment group (n = 40)	5 (12.50%)	2 (5.00%)	3 (7.50%)
Conventional treatment group (n = 40)	4 (10.00%)	2 (5.00%)	2 (5.00%)
*p*	1.000		

## Discussion

Post-ischemic stroke depression and anxiety are common complications in ischemic stroke patients. They are emotional disorders related to ischemic stroke events and are mainly manifested as low mood, reduced activity, and slowed thinking. Symptoms associated with post-stroke depression may appear within a few days to one year after a stroke, with approximately 71% of cases occurring within 3 months of onset, representing the peak period [26]. Post-ischemic stroke depression and anxiety are often accompanied by neurological deficits and frequently have insidious onset, making them easy to be overlooked in clinical practice and potentially leading to serious consequences [27]. These psychological disorders can not only reduce patients’ initiative in rehabilitation training and treatment compliance, but may also further aggravate cognitive impairment, delay the recovery of neurological function, and even increase the risk of ischemic stroke recurrence and death. Improving awareness of the typical symptoms of post-ischemic stroke depression and anxiety and promoting systematic early screening and assessment are critical for avoiding poor outcomes and improving patient prognosis [28]. For patients with aphasia, cognitive impairment, or severe physical disability, their emotional problems are more easily masked and require standardized scales and multidimensional assessments for early identification. Therefore, exploring and validating effective treatment strategies for post-ischemic stroke depression and anxiety, especially the clinical value of combined pharmacological and non-pharmacological treatment models, is of great practical significance and clinical necessity.

This study retrospectively investigated the clinical efficacy and neuroimaging changes of escitalopram combined with LF-rTMS in treating post-ischemic stroke depression and anxiety. The results showed that compared with escitalopram alone, the combined treatment was associated with greater relief of anxiety and depression, improved BR ultrasound parameters, higher overall clinical response, without a significant increase in the incidence of adverse reactions. The results of this study are consistent with previous literature reports on rTMS treatment [29, 30]. This approach may have unique advantages in promoting the normalization of BR echo structure and might achieve a multi-level therapeutic effect from symptom improvement to structural repair as an exploratory clinical observation. Feng *et al*. [31] found that LF-rTMS acting on the right prefrontal cortex can effectively alleviate post-ischemic stroke depressive symptoms. Based on this, this study further combined clinical scale assessment with objective imaging indicators to support the mood-improving effect of the combined treatment and describe corresponding structural changes in key brainstem nuclei. This study supports the synergistic effect of combined therapy in improving mood symptoms, and for the first time provides objective imaging evidence from the perspective of changes in BR structural echogenicity, describing corresponding structural variations related to mood improvement.

The clinical effect of escitalopram combined with LF-rTMS may involve multi-target regulation. Escitalopram is a highly selective 5-hydroxytryptamine (5-HT) reuptake inhibitor with a chemical structure corresponding to the S-enantiomer of citalopram, conferring higher selectivity and clinical efficacy [32]. This agent effectively inhibits 5-HT reuptake by presynaptic neurons via blocking the 5-HT transporter, thereby enhancing serotonergic neurotransmission in the synaptic cleft and exerting antidepressant effects [33, 34]. As a commonly used first-line antidepressant in clinical practice, escitalopram has shown favorable neuroprotective and functional regulatory potential while improving mood symptoms, providing a pharmacological foundation for combination therapy. LF-rTMS acts on the dorsolateral prefrontal cortex of the right prefrontal cortex, which is associated with improved mood symptoms and related changes in BR echogenicity as exploratory imaging observations [35, 36]. This non-invasive neuromodulation method can directly act on the brain network closely related to emotion regulation, potentially overcoming some limitations of medication alone and enabling complementary effects of pharmacotherapy and physical treatment. The raphe nuclei (BR) in the brainstem are the primary source of central 5-HTergic neurons; reduced or interrupted echogenicity observed in the BR via transcranial sonography (TCS) may reflect impaired function of the 5-HTergic system. Abnormal echogenicity in the BR (reduced or interrupted) is positively correlated with the severity of depression and anxiety, suggesting that changes in BR echogenicity may serve as an imaging biomarker for depression and anxiety [37, 38]. These findings collectively demonstrate the objective efficacy of the combined treatment in improving mood symptoms from a clinical perspective, and provide objective observational evidence for the synergistic effect of the combined treatment in this study.

This study provides preliminary insights into the synergistic mechanism of combined therapy. Escitalopram combined with LF-rTMS may be associated with increased concentrations of key neurotransmitters and improved functional connectivity and plasticity in related brain regions, suggesting a more comprehensive synergistic effect. Our findings are supported by several similar studies demonstrating that rTMS combined with SSRIs yields superior efficacy in post-ischemic stroke depression compared with antidepressant monotherapy [39, 40, 41]. These studies attributed the synergistic effect to enhanced modulation of synaptic neurotransmission and prefrontal-limbic network function. However, some studies indicate that the additional benefits of combined rTMS in certain functional domains (such as spasticity and cognitive impairment) remain unclear, which may be related to heterogeneity in stimulation parameters, stroke severity, lesion location, and treatment duration [42, 43]. Compared with these previous investigations, the present study offers an innovative perspective by combining clinical symptom evaluation with exploratory BR echogenicity assessment, providing preliminary imaging evidence for the central mechanism underlying combined treatment effects. This combination regimen has a good safety profile, without significantly increasing adverse reactions. The use of LF-rTMS on top of conventional treatment did not introduce new safety risks, and the synergy between drug and physical treatment did not lead to an increased incidence of adverse events, demonstrating high clinical tolerability and feasibility, supporting its potential clinical value.

It should be noted that this study has several limitations. First, this was a retrospective, non-randomized observational study with group assignment based on clinical practice rather than random allocation or matching. Consequently, we cannot exclude potential imbalances in ischemic stroke severity, lesion location, baseline psychiatric burden, or rehabilitation intensity between the two groups, which may introduce selection bias and confound the interpretation of treatment effects. Second, the single-center design and relatively small sample size limit the generalizability of the results. Third, the study included only patients with moderate-to-severe anxiety and depression, as defined by HAMD and HAMA severity criteria, and did not enroll patients with mild symptoms. Therefore, our findings may not be directly generalizable to patients with mild post-stroke anxiety or depression. In addition, the short follow-up period restricts the evaluation of long-term efficacy and recurrence risk. Furthermore, education level was classified dichotomously as junior high school or below vs. above junior high school, which represents a relatively crude grouping. Future research can further clarify its long-term effects and mechanistic details by extending the observation period, setting up sham stimulation controls, using years of education as a continuous variable in sensitivity analysis, and incorporating more neuroimaging and electrophysiological indicators.

## Conclusions

In conclusion, escitalopram combined with LF-rTMS effectively alleviates anxiety and depression in ischemic stroke patients and is associated with improved BR echogenicity as an exploratory imaging finding. This combined regimen is well tolerated and safe, and may serve as a potential and promising option for post-ischemic stroke depression and anxiety. Future prospective, randomized, sham-controlled studies with larger samples and longer follow-up are warranted to confirm its long-term efficacy and safety.

## Availability of Data and Materials

The datasets used and/or analyzed during the current study are available from the corresponding author on reasonable request.
